# Online‐based Recruitment Methods for Community‐Dwelling Older Adults: Challenges and Strategies

**DOI:** 10.1002/alz.093018

**Published:** 2025-01-09

**Authors:** Hae‐Ra Han, Deborah Min

**Affiliations:** ^1^ Johns Hopkins University School of Nursing, Baltimore, MD USA; ^2^ New York University, New York, NY USA

## Abstract

**Background:**

Despite technology adoption and increased dependence on digital technologies, the digital divide persists among older adults. The purpose of this study was to understand barriers and facilitators of recruiting non‐English speaking older individuals who are cognitively impaired along with their caregivers for PLAN, an ongoing RCT designed to promote the transition of Korean American older adults with probable dementia and their caregivers into the healthcare system for adequate diagnostic follow‐up and care. We also examined online‐based recruitment strategies focused on older adults reported in relevant published studies to compare with our experiences.

**Method:**

Data sources included recruitment tracking files, study team meeting minutes, and interviews with community staff and consultants. We also conducted a review of published studies that used online‐based recruit of community‐dwelling older adults.

**Result:**

Five themes emerged: unfamiliarity with technology—limited digital literacy; differences in internet access and use across older age groups; providing technological support to promote recruitment; successful and unsuccessful recruitment using social media; and other diverse online‐based methods. Direct quotes from multiple sources for the PLAN trial revealed technological challenges that were common among immigrant older adults. While the studies included in the review indicated similar internet accessibility and use between young‐old and older‐old adults, actual online recruitment was least successful in the older‐old group. Most studies included in the review used social media but the success varied across these studies with slightly more than half of the studies reporting little to no success. Similarly, other online‐based methods such as e‐newsletters, emails, or postings on professional organizational websites yielded varying successes.

**Conclusion:**

Our analysis revealed the digital divide along with the issue of limited digital literacy in the recruitment of older adults, particularly non‐English speaking immigrants. The usefulness of online‐based recruitment of older adults is uncertain due, in large part, to limited sociodemographic diversity noted in the samples included in the review. Future research needs to explore the role of race/ethnicity and other characteristics such as socioeconomic status, gender, education, access to technology, and digital literacy in relation to online‐based recruitment for adequate representation of diverse older adults in research.

